# MicroRNA-221-3p alleviates cell apoptosis and inflammatory response by targeting cyclin dependent kinase inhibitor 1B in chronic obstructive pulmonary disease

**DOI:** 10.1080/21655979.2021.1967837

**Published:** 2021-09-13

**Authors:** Hua Yang, Lijuan Zhang, Quandong Wang

**Affiliations:** Department of Gerontology, The First People’s Hospital of Lianyungang, Xuzhou Medical University Affiliated Hospital of Lianyungang, Lianyungang, Jiangsu, China

**Keywords:** Mir-221-3p, CDKN1B, chronic obstructive pulmonary disease, 6HBE cells, apoptosis, inflammatory response

## Abstract

As a chronic bronchitis or emphysema featured by airflow obstruction, chronic obstructive pulmonary disease (COPD) can further develop into respiratory failure and pulmonary heart diseases. MicroRNAs (miRNAs) are crucial mediators in COPD. Nevertheless, the specific role and molecular mechanism of microRNA-221-3p (miR-221-3p) in COPD are unclear. This research aimed to probe into the role of miR-221-3p in COPD. Bioinformatics analysis and a series of assays including western blot, luciferase reporter, reverse transcription quantitative polymerase chain reaction, flow cytometry, cell counting kit-8 and enzyme linked immunosorbent assay were used to explore the functions and mechanism of miR-221-3p in COPD. First, miR-221-3p level was validated to be lowly expressed in the lung tissues of COPD patients and 16HBE cells stimulated by cigarette smoke extract (CSE). Functionally, miR-221-3p overexpression inhibited inflammatory response and apoptosis in CSE-treated 16HBE cells. Moreover, we predicted 5 potential targets of miR-221-3p and found that miR-221-3p shared binding site with cyclin dependent kinase inhibitor 1B (CDKN1B). CDKN1B was targeted by miR-221-3p in CSE-treated 16HBE cells. CDKN1B was negatively modulated by miR-221-3p. Finally, rescue experiments demonstrated that overexpressed CDKN1B counteracted the influences of miR-221-3p on apoptosis and inflammatory response in CSE-treated 16HBE cells. Our data showed that miR-221-3p alleviated cell apoptosis and inflammatory response via targeting CDKN1B in an *in vitro* model of COPD.

## 1. Introduction

Chronic obstructive pulmonary disease (COPD) is a common disease with persistent airflow restriction [1–3]. The progression of airflow restriction is associated with the increase of chronic inflammatory response of airway and lung to toxic particles or gases [4,5]. Among all the risk factors for COPD, cigarette smoking is considered the commonest risk factor [6,7]. The disability and mortality resulting from COPD are very high and the incidence rate of this disease in people over 40 years old has reached 9% to 10% [8,9]. Airway remodeling is a critical feature of COPD and is characterized by the aberrant repair of the epithelium [10]. Bronchial epithelial cells are the first anatomical barrier exposed to noxious gases and particles of cigarette smoke, which can initiate airway remodeling in COPD [11]. COPD is a preventable and treatable disease, but is difficult to be diagnosed in patients with mild disease, and an improved understanding of the underlying mechanism of this disease is necessary for the diagnosis and treatment of this disease.

MicroRNAs (miRNAs) are short noncoding RNAs consisting of 19–25 nucleotides, and they are reported to be involved in diverse diseases [12–14]. Notably, miRNAs are reported to be involved in COPD. For example, miR-212-5p exerts a protective effect on COPD [15]. MiR-543 modulates COPD progression by targeting interleukin-33 [16]. MiR-34 c-5p exerts protective effect in COPD via regulating C-C motif chemokine ligand 22 [17]. Previously, the roles of microRNA-221-3p (miR-221-3p) are studied in several diseases [18–21]. Importantly, miR-221-3p regulates several pulmonary disorders [19,22–26]. For example, lncRNA taurine up-regulated 1 offsets lipopolysaccharide-induced inflammation and cell apoptosis of macrophage [27]. Reduced miR-221-3p level is related to airway eosinophilic inflammatory response in asthma [19]. Particularly, it has been found that miR-221-3p is downregulated in the lung tissues of COPD patients [28]. Additionally, previous studies indicated that miRNAs negatively modulate gene levels via interacting with the 3ʹuntranslated regions (3ʹUTRs) of target messenger RNAs (mRNAs) post-transcriptionally [29,30]. MiR-221-3p was indicated to regulate some human diseases by targeting specific mRNAs. MiR-221-3p modulates the microvascular dysfunctions in diabetic retinopathy via regulating TIMP metallopeptidase inhibitor 3 [31]. MiR-221-3p targets phosphoinositide-3-kinase regulatory subunit 1 to facilitate migration and tube formation abilities of human umbilical vein endothelial cells [32]. MiR-221-3p modulates airway eosinophilic inflammatory response via targeting C-X-C motif chemokine ligand 17 in asthma [19]. Nonetheless, the regulatory mechanism of miR-221-3p in CSE-treated 16HBE cells was unclear.

Cigarette smoke extract (CSE) was commonly used to treat human bronchial epithelial 16HBE cells to mimic COPD *in vitro* in the previous studies [33–35]. In this research, we hypothesized that miR-221-3p can regulate CSE-stimulated 16HBE cell injury. The aim of this study was to investigate the biological role and molecular regulatory mechanism of miR-221-3p in CSE-treated 16HBE cells. Our research may deepen our understanding of the pathogenesis of COPD and point out the potential of miR-221-3p in the treatment of COPD.

## 2. Materials and methods

### 2.1 Tissue samples

The lung tissues were acquired from 48 participants (11 nonsmokers, 21 smokers with COPD and 16 smokers without COPD) at The First People’s Hospital of Lianyungang, Xuzhou Medical University Affiliated Hospital of Lianyungang. All participants signed the informed consents. This study was conducted in accordance with the Declaration of Helsinki and the approval was obtained from the Ethics Committee of The First People’s Hospital of Lianyungang, Xuzhou Medical University Affiliated Hospital of Lianyungang.

### 2.2 Cell lines and cell culture

16HBE is a SV40 large T antigen-transformed human bronchial epithelial cell line that retains the differentiated morphology and functions of normal airway epithelial cells. The 16HBE cells were purchased from the American Type Culture Collection (ATCC; USA). Dulbecco’s modified Eagle’s medium USA) with 10% fetal bovine serum (Sigma-Aldrich, USA), 100 μg/ml streptomycin (Sigma-Aldrich, USA) and 100 U/ml penicillin (Sigma-Aldrich, USA) was employed to incubate the cells. Next, the cells were cultured in the humidified incubator with 5% CO_2_ at 37°C.

### 2.3 Preparation of CSE

Ten cigarettes (Yuxi, Hongta Cigarette Company, Yunnan, China) were used to obtain the smoke. The obtained smoke was bubbled using 25 ml of media. Next, the collected suspension was titrated to pH 7.4, filter-sterilized, and considered to be 100% CSE. After that, the CSE sample was diluted using phosphate buffer solution to the concentrations of 1%, 2% and 3%, and the stock CSE was kept at −80°C.

### 2.4 Cell transfection

MiR-221-3p was overexpressed using miR-221-3p mimics and negative control (NC) mimics served as corresponding negative controls. The full-length sequence of cyclin dependent kinase inhibitor 1B (CDKN1B) was synthesized and subcloned in the pcDNA3.1 (GenePharma, Shanghai, China) plasmid to generate the pcDNA3.1/CDKN1B. All plasmids (GenePharma, Shanghai, China) were transfected into 16HBE cells by Lipofectamine 2000 (Invitrogen, USA) and the transfection continued for 48 h.

### 2.5 Reverse transcription quantitative polymerase chain reaction (RT-qPCR)

TRIzol reagent (Invitrogen, USA) was employed to isolate the total RNA from 16HBE cells. The extracted RNA was then reverse transcribed into cDNA adopting a Reverse Transcription Kit (Takara, Dalian, China). SYBR Premix Ex Taq (Takara, Dalian, China) was used for RT-qPCR. The reaction was performed on an Applied Biosystems 7000 Sequence Detection System (Applied Biosystems). The detection results for mRNAs were normalized to glyceraldehyde-3-phosphate dehydrogenase (GAPDH). U6 acted as the endogenous reference for miRNAs. Expression fold changes were calculated using the 2^−ΔΔCt^ method [36].

### 2.6 Western blot

Radio Immunoprecipitation Assay lysis buffer was employed to lyse 16HBE cells. Next, the collected protein sample was isolated using sodium dodecyl sulfate polyacrylamide gel electrophoresis, transferred to polyvinylidene fluoride membranes, and blocked by 5% skim milk powder for 1 h. Next, primary antibodies were added into the membranes at 4°C overnight. Primary antibodies include antibodies against B cell leukemia/lymphoma 2 (abbreviated as Bcl-2; ab182858, Abcam), cleaved-caspase-3 (ab32042, Abcam), Bcl2 associated X, apoptosis regulator (abbreviated as Bax; ab32503, Abcam), CDKN1B (ab32034, Abcam) and GAPDH (ab8245, Abcam). After the membranes were incubated with the secondary antibodies at 37°C for 1 h, an ECL Plus reagent (Applygen Technologies Inc., Beijing, China) was employed to develop the bands.

### 2.7 Luciferase reporter assay

The wild type or mutant sequence of CDKN1B 3ʹUTR was subcloned into the pmirGLO reporters (Promega, USA), and named CDKN1B-Wt or CDKN1B-Mut, respectively. Next, CDKN1B-Mut or CDKN1B-Wt was cotransfected with NC mimics or miR-221-3p mimics in 16HBE cells. Lipofectamine 2000 was employed to perform the transfection. After 48 h of transfection, a dual-luciferase reporter assay system kit (Promega) was used to conduct the luciferase assay. Modulus single-tube multimode reader (Promega) was used to detect luciferase activity.

### 2.8 CCK-8 assay

16HBE cell viability was detected using a Cell Counting Kit-8 (CCK-8). The transfected cells at the density of 1 × 10^4^ cells/well were plated onto 96-well plates. After culture for 0 h, 12 h, 24 h, 36 h and 48 h, CCK-8 solution (10 μl) was supplemented into each well. After that, the incubation continued for 4 h. Cell viability was detected using the microplate reader (EL340; BioTek Instruments, Hopkinton, MA) at 450 nm.

### 2.9 Flow cytometry analysis

The flow cytometry was performed to assess 16HBE cell apoptosis with an Annexin V Kit (Beyotime, China) with reference to protocols stipulated by the manufacturer. Cells transfected with indicated plasmids or negative controls were collected 48 h later and washed twice with PBS. Subsequently, cells (1 × 10^6^ cells/mL) were double stained using propidium iodide (PI) and Annexin V-FITC. A BD FACSCalibur flow cytometry was used to analyze cell apoptosis on the CELLQUEST program (Becton Dickinson, USA).

### 2.10 Enzyme-linked immunosorbent assay (ELISA)

Culture supernatant was collected, and ELISA kits (R&D Systems, USA) were used to measure the concentrations of pro-inflammatory cytokines cytochrome c oxidase subunit II (COX-2), tumor necrosis factor alpha (TNF-α), interleukin 1 beta (IL-1β) and interleukin 6 (IL-6) in line with manufacturer’s instructions.

### 2.11 Data statistics

Data analysis was conducted employing SPSS 23.0 (IBM SPSS, Chicago, IL, USA). All data are exhibited as the means ± standard deviation. A one-way analysis of variance followed by HolmSidak pos-hoc test was performed to analyze the statistical significance in three or more groups. Difference between two groups was assessed using Student’s t-test. Pearson correlation analysis was used to analyze the correlation between mRNA expression and miRNA expression. P-value of < 0.05 was regarded statistically significant.

## 3. Results

### 3.1 MiR-221-3p was lowly expressed in lung tissues of COPD patients and CSE-treated 16HBE cells

**Figure 1.**
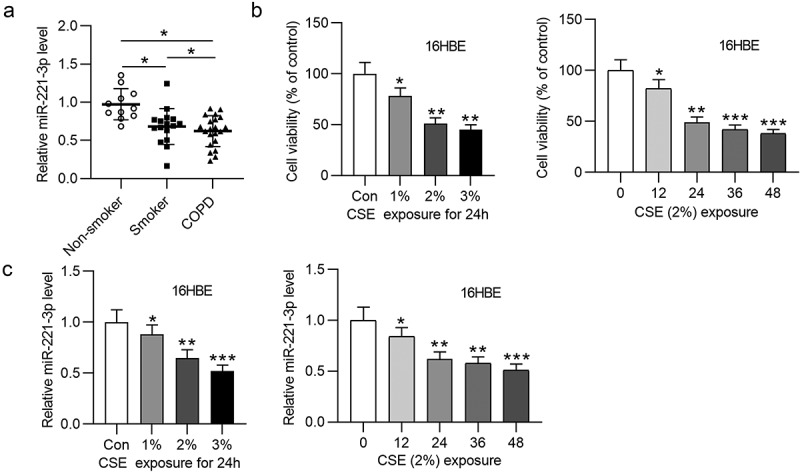
MiR-221-3p exhibits a low expression in COPD tissues and 16HBE cells. (a) MiR-221-3p levels in the tissues of nonsmokers (n = 11), smokers without COPD (n = 16) and smokers with COPD (n = 21) were detected by RT-qPCR. (b) The viability of 16HBE cells in response to different concentrations (0%, 1%, 2%, 3%) of CSE for 24 h (the left panel) or in response to 2% CSE for 0 h, 12 h, 24 h, 36 h, 48 h (the right panel) was detected by CCK-8. (c) MiR-221-3p levels in 16HBE cells by treatment of different concentrations (0%, 1%, 2%, 3%) of CSE for 24 h (the left panel) or by 2% CSE for 0 h, 12 h, 24 h, 36 h, 48 h (the right panel) was detected by RT-qPCR. *P < 0.05, **P < 0.01, ***P < 0.001

To identify the role of miR-221-3p in COPD, miR-221-3p level in COPD was detected. RT-qPCR analysis suggested that miR-221-3p level was lower in lung tissues of smokers and COPD patients relative to nonsmokers (Figure 1a). Next, we treated 16HBE cells by different time or concentrations. In comparison with the control group, 16HBE cell viability was dose-dependently decreased by 1%, 2% and 3% CSE (Figure 1b). Subsequently, 16HBE cells were exposed to 2% CSE for 0 h, 12 h, 24 h, 36 h and 48 h, and we observed that the viability of 16HBE cells was time-dependently decreased by 2%CSE stimulation (Figure 1b). Next, we evaluated the influence of CSE on miR-221-3p level. Our findings demonstrated that in comparison with the control group, miR-221-3p level was concentration-dependently and time-dependently reduced by CSE in 16HBE cells (Figure 1c). In summary, the above data showed that miR-221-3p presents a low level in lung tissues of COPD patients and CSE- stimulated 16HBE cells.

### 3.2 MiR-221-3p overexpression inhibited cell apoptosis and inflammation in 16HBE cells

**Figure 2.**
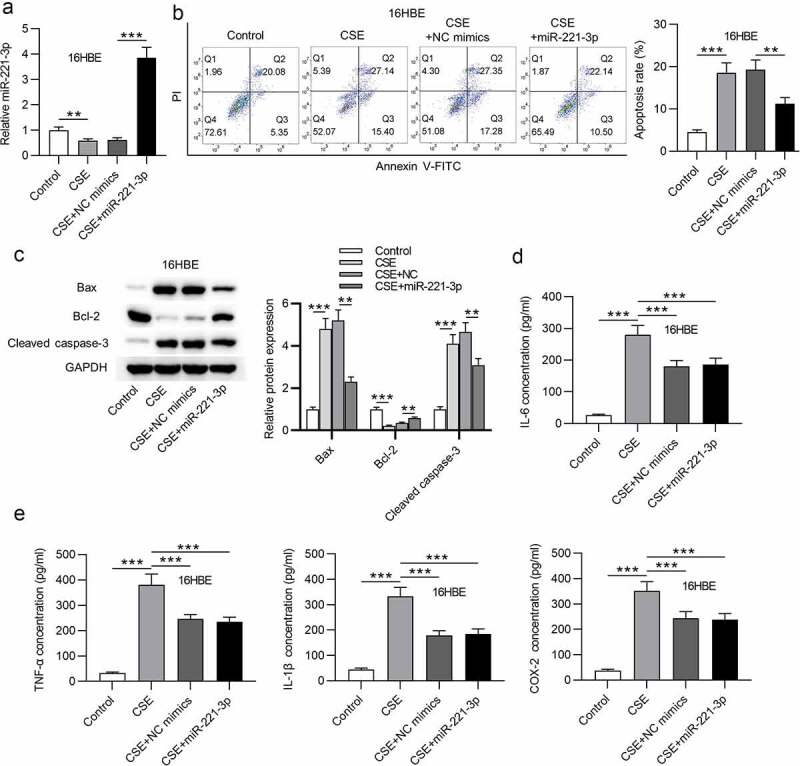
Overexpressed miR-221-3p suppressed the apoptosis and inflammation of 16HBE cells. (a) MiR-221-3p expression in the four groups. (b) The apoptosis of 16HBE cells in different groups. (c) The protein levels of Bax, cleaved caspase-3 and Bcl-2 in 16HBE cells from different groups. (d-e) The concentrations of inflammatory cytokines COX-2, IL-6, TNF-α and IL-1β in the four groups. **P < 0.01, ***P < 0.001

**Figure 3.**
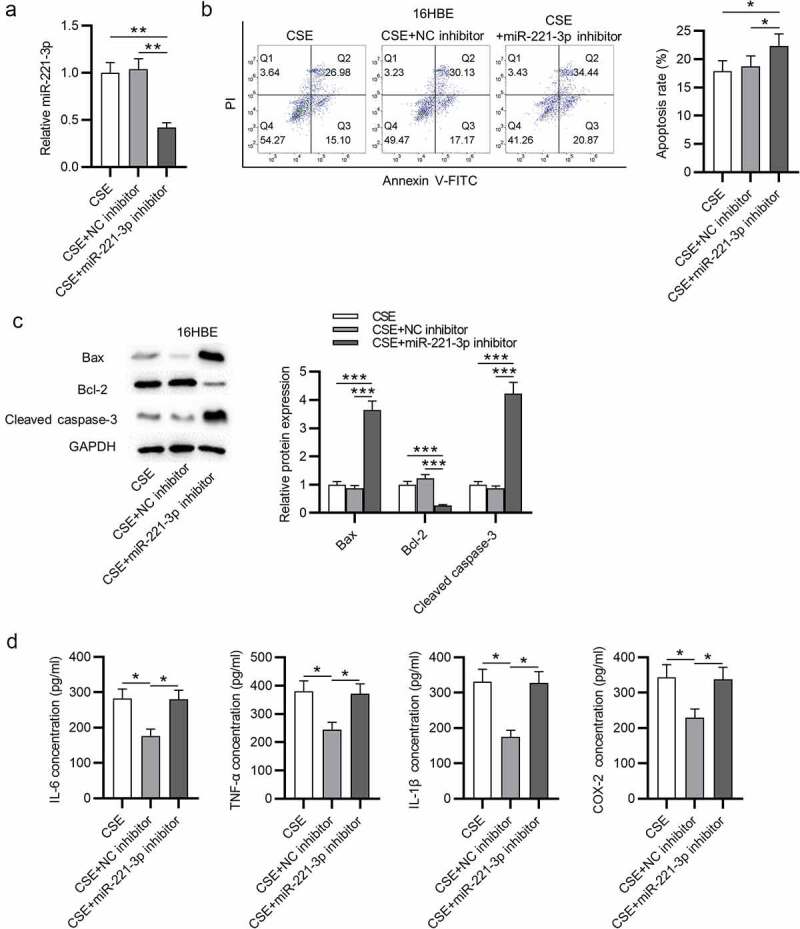
Overexpressed miR-221-3p suppressed the apoptosis and inflammation of 16HBE cells. (a) MiR-221-3p silencing efficiency was detected by RT-qPCR. (b) The apoptosis of 16HBE cells under the condition of miR-221-3p deficiency was detected by CCK-8. (c) The protein levels of Bax, cleaved caspase-3 and Bcl-2 in 16HBE cells under the condition of miR-221-3p deficiency were detected by western blotting. (d) The concentrations of inflammatory cytokines COX-2, IL-6, TNF-α and IL-1β under the condition of miR-221-3p deficiency were detected by ELISA. *P < 0.05, **P < 0.01, ***P < 0.001

Subsequently, the functions of miR-221-3p in apoptosis and inflammatory response of 16HBE cells were investigated. First, we overexpressed miR-221-3p by transfection of miR-221-3p mimics into 16HBE cells (Figure 2a). Next, we found that CSE promoted 16HBE cell apoptosis, which was then offset by miR-221-3p mimics (Figure 2b). Additionally, Bax and cleaved caspase-3 protein levels were increased in CSE-treated 16HBE cells, and miR-221-3p mimics decreased cleaved caspase-3 and Bax protein levels; Bcl-2 protein level was reduced in CSE-treated 16HBE cells, and miR-221-3p mimics improved Bcl-2 protein level (Figure 2c). Moreover, we observed that the concentrations of inflammatory cytokines COX-2, IL-1β, TNF-α and IL-6 were enhanced in 16HBE cells after treatment of CSE, while the concentrations were then reduced upon miR-221-3p mimics (Figure 2d-e). The above data suggested that miR-221-3p restrained the apoptosis and inflammatory response of CSE-stimulated 16HBE cells. Furthermore, miR-221-3p inhibitor was transfected into the 16HBE cells. The silencing efficiency of miR-221-3p was verified by RT-qPCR (Figure 3a). MiR-221-3p knockdown promoted the apoptosis and inflammatory response of CSE-stimulated 16HBE cells (Figure 3b-d).

### 3.3 CDKN1B was targeted by miR-221-3p

**Figure 4.**
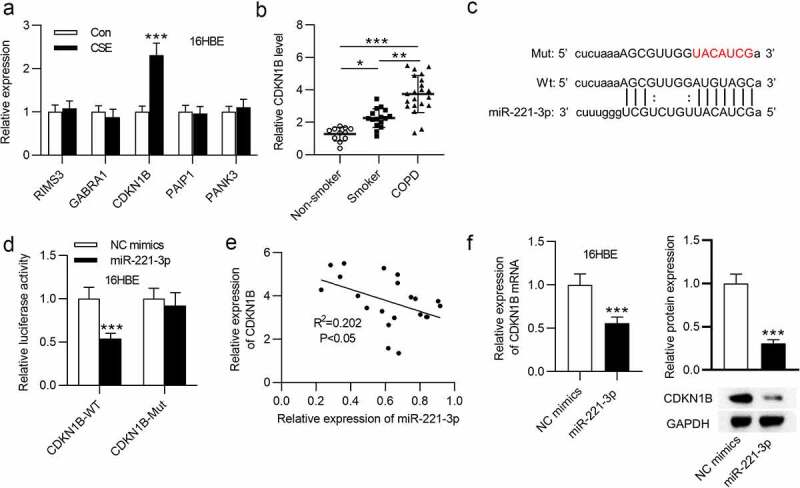
CDKN1B was targeted by miR-221-3p. (a) The expressions of the 5 mRNAs that shared binding site with miR-221-3p in 16HBE cells with or without CSE stimulation. (b) CDKN1B expression in the tissues of nonsmokers (n = 11), smokers without COPD (n = 16) and smokers with COPD (n = 21). (c) The binding site of CDKN1B 3ʹUTR with miR-221-3p. (d) The binding capability of CDKN1B with miR-221-3p was assessed by luciferase reporter assay. (e) The correlation of CDKN1B level and miR-221-3p level in 21 COPD tissues was determined using Pearson correlation analysis. (f) Effects of miR-221-3p on CDKN1B mRNA and protein levels were detected by RT-qPCR and western blotting. *P < 0.05, **P < 0.01, ***P < 0.001

Subsequently, the potential targets of miR-221-3p in CSE-stimulated 16HBE cells were explored. First, from starBase database (http://starbase.sysu.edu.cn), we identified 5 mRNAs binding with miR-221-3p. RT-qPCR analysis showed that CDKN1B was highly expressed in CSE-treated 16HBE cells (Figure 4a). Additionally, CDKN1B exhibited a higher level in the lung tissues of smokers and COPD patients than nonsmokers (Figure 4b). Next, we evaluated the binding of CDKN1B with miR-221-3p. The binding site of CDKN1B 3ʹUTR with miR-221-3p was exhibited in Figure 4c. Luciferase activity of the CDKN1B-Wt reporter was weakened by miR-221-3p upregulation, but the luciferase activity of the CDKN1B-Mut reporter presented no significant change (Figure 4d). Next, we observed that miR-221-3p overexpression was in a negative correlation with CDKN1B expression in 21 COPD tissues (Figure 4e). Furthermore, miR-221-3p overexpression reduced CDKN1B mRNA and protein levels in 16HBE cells (figure 4f). In summary, CDKN1B was targeted by miR-221-3p in 16HBE cells.

### 3.4 CDKN1B overexpression offset the influence of miR-221-3p on cell apoptosis and inflammatory response in 16HBE cells

**Figure 5.**
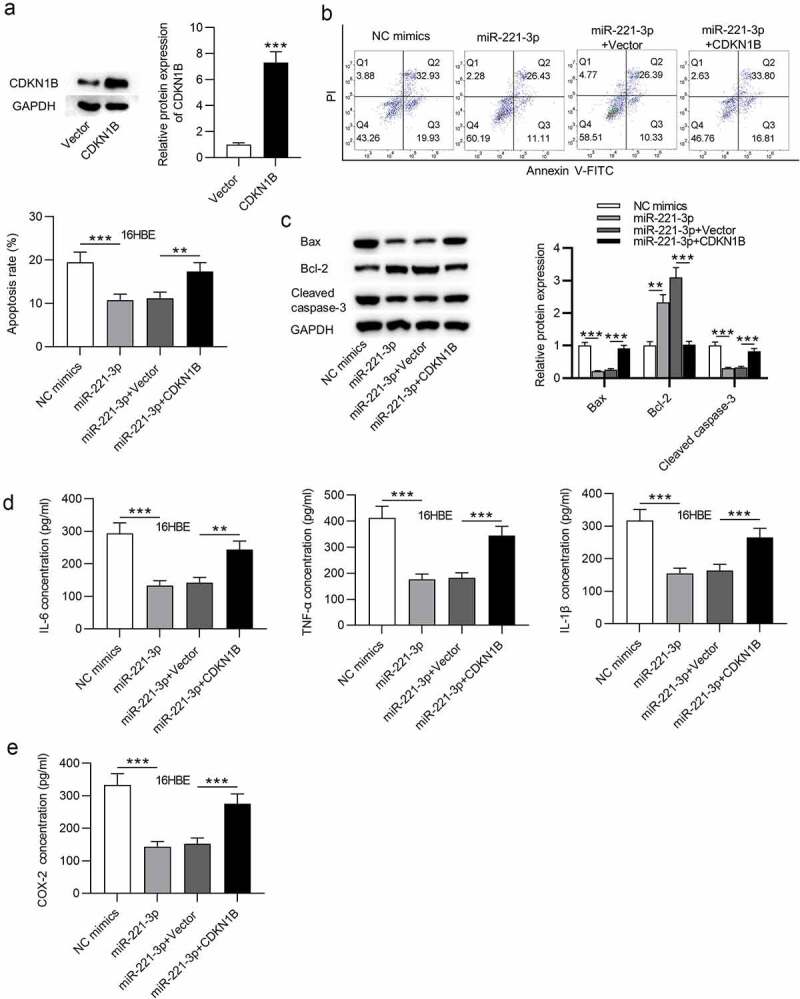
CDKN1B overexpression counteracted the influence of miR-221-3p on the apoptosis and inflammation of 16HBE cells. (a) The overexpression efficiency of CDKN1B was detected by western blotting. (b) 16HBE cell apoptosis with transfection of indicated plasmids was detected by annexin V-FITC/PI staining and analyzed by flow cytometry. (c) The protein levels of Bax, Bcl-2 and Cleaved Caspase-3 in 16HBE cells from different groups were detected by western blotting. (d-e) The concentrations of inflammatory cytokines COX-2, IL-6, TNF-α and IL-1β in the four groups were detected by ELISA. **P < 0.01, ***P < 0.001

Finally, to validate whether miR-221-3p modulates cell apoptosis and inflammatory response in CSE-stimulated 16HBE cells via CDKN1B, rescue assays were performed. First, CDKN1B was overexpressed by transfection of pcDNA3.1/CDKN1B into CSE-stimulated 16HBE cells (Figure 5a). Next, flow cytometric analysis revealed that CDKN1B upregulation partially offset the suppressive influence of miR-221-3p mimics on the apoptosis of 16HBE cells (Figure 5b). Additionally, CDKN1B overexpression partially restored the decline of cleaved caspase-3 and Bax protein levels and the increase of Bcl-2 protein levels resulting from miR-221-3p mimics (Figure 5c). Moreover, we observed that CDKN1B overexpression partially rescued the miR-221-3p mimics-induced decrease of inflammatory cytokines COX-2, IL-1β, IL-6, and TNF-α (Figure 5d-e). In conclusion, the above data suggested that miR-221-3p regulates cell apoptosis and inflammatory response in CSE-stimulated 16HBE cells via CDKN1B.

## 4. Discussion

Previously, aberrant expression of miRNAs has been reported to be related to several pulmonary disorders [15,37,38]. In the current research, we probed the biological function and molecular regulatory mechanism of miR-221-3p in CSE-stimulated 16HBE cells, an *in vitro* model of COPD. Our data suggested that miR-221-3p alleviated cell apoptosis and inflammatory response via regulating CDKN1B in CSE-stimulated 16HBE cells.

We evaluated miR-221-3p levels in lung tissues of smokers and found a significant downregulation of miR-221-3p in smokers compared with the nonsmokers, and in smokers with COPD compared with the smokers without COPD. Consistently, according to a previous research, miR-221-3p expression was in a low level in some diseases. Expression of miR-221-3p is indicated to be low in cartilage tissues of patients with osteoarthritis [39]. MiR-221-3p expression is downregulated in peripheral nerve injury [20] and preeclampsia [40]. Notably, miR-221-3p is reported to be lowly expressed in lung tissues of COPD patients [28]. Contradictorily, Yahui Shen *et al*. demonstrated that serum levels of miR-221-3p were higher in 155 COPD patients than 77 healthy volunteers [41]. The contradiction of miR-221-3p expression in COPD patients might be associated with sample size and individual variation of patients.

Subsequently, the investigation of the functions of miR-221-3p in COPD was conducted. Previous studies have indicated that miR-221-3p regulates cell apoptosis and inflammatory response in some diseases [27,42,43]. Reduced miR-221-3p might alleviate airway eosinophilic inflammatory response in asthma [19]. Upregulated miR-221-3p exerts an anti-inflammatory effect in coronary heart disease [44]. MiR-221-3p alleviates oxidized low-density lipoprotein-induced apoptosis and inflammation in atherosclerosis [43]. In our study, miR-221-3p overexpression restrained inflammatory response and cell apoptosis in CSE-stimulated 16HBE cells.

Additionally, miR-221-3p was indicated to regulate some human diseases by targeting specific mRNAs [19,23,31]. However, whether miR-221-3p regulates COPD in the same manner remains ambiguous. CDKN1B, also known as CDKN4, KIP1, MEN1B, MEN4 and P27KIP1, has been reported to be an active regulator in many biological processes [45,46]. Mechanistically, CDKN1B was reported to act as a downstream target of miRNAs in many diseases. MiR-142-3p facilitates cell viability and suppresses cell apoptosis via regulating CDKN1B after sciatic nerve injury [47]. CDKN1B regulates oxyhemoglobin-induced neuronal cell apoptosis and inflammatory responses via miR-502-5p following subarachnoid hemorrhage [48]. An exploration of the regulatory mechanism of miR-221-3p in the present study revealed that CDKN1B 3ʹUTR was targeted and degraded by miR-221-3p. CDKN1B expression was higher in CSE-stimulated 16HBE cells and in COPD patients. Overexpressed CDKN1B partially countervailed the suppressive influence of miR-221-3p on cell apoptosis and inflammatory response in CSE-treated 16HBE cells, indicating that CDKN1B is a positive regulator in COPD and is involved in miR-221-3p-medaited COPD pathogenesis. Considering the partial rescue effects of CDKN1B on miR-221-3p, it can be inferred that some other modulators associated with the miR-221-3p/CDKN1B axis regulate 16HBE cell apoptosis and inflammatory response and further mediate COPD, which can explain the large difference in CDKN1B expression despite the relatively small difference in miR-221-3p expression in COPD patients compared to control individuals.

There are some limitations of the present study. First, 16HBE is a transformed cell line and cannot completely imitate human bronchial epithelial cell line. Furthermore, we did not investigate other factors, such as epithelial-mesenchymal transition and oxidative stress that are involved in bronchial remodeling after CSE treatment. It is not clear whether miR-221-3p plays a protective role in airway remodeling in animal models, which remains to be investigated in future study.

## 5. Conclusions

This research manifested that miR-221-3p alleviated cell apoptosis and inflammatory response via targeting CDKN1B in CSE-treated 16HBE cells. Our finding disclosed the potential molecular mechanism of miR-221-3p in COPD, and our finding exhibited the potential of miR-221-3p in COPD treatment.

## References

[1] Curtis DJ, Smale A, Thien F, et al. Chronic airflow obstruction in long-term survivors of allogeneic bone marrow transplantation. Bone Marrow Transplant. 1995 Jul;16(1):169–173. 7581118

[2] O’Donnell DE, Gebke KB. Examining the role of activity, exercise, and pharmacology in mild COPD. Postgrad Med. 2014 Sep;126(5):135–145. 10.3810/pgm.2014.09.2808 25295658

[3] Spero K, Bayasi G, Beaudry L, et al. Overdiagnosis of COPD in hospitalized patients. Int J Chron Obstruct Pulmon Dis. 2017;12:2417–2423. 28860736 10.2147/COPD.S139919 PMC5565250

[4] Brandsma CA, Van den Berge M, Hackett TL, et al. Recent advances in chronic obstructive pulmonary disease pathogenesis: from disease mechanisms to precision medicine. J Pathol. 2020 Apr;250(5):624–635. 31691283 10.1002/path.5364 PMC7216938

[5] Hogg JC, Timens W. The pathology of chronic obstructive pulmonary disease. Annu Rev Pathol. 2009;4:435–459. 18954287 10.1146/annurev.pathol.4.110807.092145

[6] Barnes PJ, Burney PG, Silverman EK, et al. Chronic obstructive pulmonary disease. Nat Rev Dis Primers. 2015 Dec;3(1):15076. 10.1038/nrdp.2015.76 27189863

[7] Duffy SP, Criner GJ. Chronic obstructive pulmonary disease: evaluation and management. Med Clin North Am. 2019 May;103(3):453–461. 30955513 10.1016/j.mcna.2018.12.005

[8] Soriano JB, Abajobir AA, Abate KH. Global, regional, and national deaths, prevalence, disability-adjusted life years, and years lived with disability for chronic obstructive pulmonary disease and asthma, 1990-2015: a systematic analysis for the global burden of disease study 2015. Lancet Respir Med. 2017 Sep;5(9):691–706. 28822787 10.1016/S2213-2600(17)30293-X PMC5573769

[9] Garvey C, Bayles MP, Hamm LF, et al. Pulmonary rehabilitation exercise prescription in chronic obstructive pulmonary disease: review of selected guidelines: an official statement from the American association of cardiovascular and pulmonary rehabilitation. J Cardiopulm Rehabil Prev. 2016 Mar-Apr;36(2):75–83. 26906147 10.1097/HCR.0000000000000171

[10] Ito JT, Lourenço JD, Righetti RF, et al. Extracellular matrix component remodeling in respiratory diseases: what has been found in clinical and experimental studies? Cells. 2019 Apr 11;8(4). DOI:10.3390/cells8040342. PMC6523091 30979017

[11] Milara J, Peiró T, Serrano A, et al. Epithelial to mesenchymal transition is increased in patients with COPD and induced by cigarette smoke. Thorax. 2013 May;68(5):410–420. 23299965 10.1136/thoraxjnl-2012-201761

[12] García-Sancha N, Corchado-Cobos R, Pérez-Losada J, et al. MicroRNA dysregulation in cutaneous squamous cell carcinoma. Int J Mol Sci. 2019 May 2;20(9). DOI:10.3390/ijms20092181. PMC6540078 31052530

[13] Giudice A, Montella M, Boccellino M, et al. Epigenetic changes induced by green tea catechins a re associated with prostate cancer. Curr Mol Med. 2017;17(6):405–420. 29256350 10.2174/1566524018666171219101937

[14] Wang J, Liu S, Shi J, et al. The role of miRNA in the diagnosis, prognosis, and treatment of osteosarcoma. Cancer Biother Radiopharm. 2019 Dec;34(10):605–613. 31674804 10.1089/cbr.2019.2939

[15] Jia Q, Chang J, Hong Q, et al. MiR-212-5p exerts a protective effect in chronic obstructive pulmonary disease. Discov Med. 2018 Nov;26(144):173–183. 30695677

[16] He H, Wang H, Pei F, et al. MiR-543 regulates the development of chronic obstructive pulmonary disease by targeting interleukin-33. Clin Lab. 2018 Jul 1;64(7):1199–1205. 30146845 10.7754/Clin.Lab.2018.180205

[17] Gao HX, Su Y, Zhang AL, et al. MiR-34c-5p plays a protective role in chronic obstructive pulmonary disease via targeting CCL22. Exp Lung Res. 2019 Feb-Mar;45(1–2):1–12. 31032652 10.1080/01902148.2018.1563925

[18] Feng J, Wang M, Li M, et al. Serum miR-221-3p as a new potential biomarker for depressed mood in perioperative patients. Brain Res. 2019 Oct 1;1720:146296. 31211948 10.1016/j.brainres.2019.06.015

[19] Zhang K, Liang Y, Feng Y, et al. Decreased epithelial and sputum miR-221-3p associates with airway eosinophilic inflammation and CXCL17 expression in asthma. Am J Physiol Lung Cell Mol Physiol. 2018 Aug 1;315(2):L253–l264. 29644894 10.1152/ajplung.00567.2017

[20] Zhao L, Yuan Y, Li P, et al. miR-221-3p inhibits schwann cell myelination. Neuroscience. 2018 May 21;379:239–245. 29577996 10.1016/j.neuroscience.2018.03.019

[21] Zhou CF, Ma J, Huang L, et al. Cervical squamous cell carcinoma-secreted exosomal miR-221-3p promotes lymphangiogenesis and lymphatic metastasis by targeting VASH1. Oncogene. 2019 Feb;38(8):1256–1268. 30254211 10.1038/s41388-018-0511-x PMC6363643

[22] Joshi SR, Dhagia V, Gairhe S, et al. MicroRNA-140 is elevated and mitofusin-1 is downregulated in the right ventricle of the Sugen5416/hypoxia/normoxia model of pulmonary arterial hypertension. Am J Physiol Heart Circ Physiol. 2016 Sep 1;311(3):H689–98. 27422986 10.1152/ajpheart.00264.2016 PMC7199238

[23] Nie X, Chen Y, Tan J, et al. MicroRNA-221-3p promotes pulmonary artery smooth muscle cells proliferation by targeting AXIN2 during pulmonary arterial hypertension. Vascul Pharmacol. 2019 May;116:24–35. 28694128 10.1016/j.vph.2017.07.002

[24] Yin G, Zhang B, Li J. miR‑221‑3p promotes the cell growth of non‑small cell lung cancer by targeting p27. Mol Med Rep. 2019 Jul;20(1):604–612. 31180541 10.3892/mmr.2019.10291 PMC6580017

[25] Yu H, Xu L, Liu Z, et al. Circ_MDM2_000139, Circ_ATF2_001418, Circ_CDC25C_002079, and Circ_BIRC6_001271 are involved in the functions of XAV939 in non-small cell lung cancer. Can Respir J. 2019;2019:9107806. 31885751 10.1155/2019/9107806 PMC6900950

[26] Zhou X, Wen W, Shan X, et al. A six-microRNA panel in plasma was identified as a potential biomarker for lung adenocarcinoma diagnosis. Oncotarget. 2017 Jan 24;8(4):6513–6525. 28036284 10.18632/oncotarget.14311 PMC5351649

[27] Hu L, Ye H, Liao J. LncRNA TUG1 reverses LPS-induced cell apoptosis and inflammation of macrophage via targeting MiR-221-3p/SPRED2 axis. Biosci Biotechnol Biochem. 2020 Aug;25:1–8. 10.1080/09168451.2020.1806704 32841583

[28] Conickx G, Mestdagh P, Avila Cobos F, et al. MicroRNA profiling reveals a role for microRNA-218-5p in the pathogenesis of chronic obstructive pulmonary disease. Am J Respir Crit Care Med. 2017 Jan 1;195(1):43–56. 27409149 10.1164/rccm.201506-1182OC

[29] Friedman RC, Farh KK, Burge CB, et al. Most mammalian mRNAs are conserved targets of microRNAs. Genome Res. 2009 Jan;19(1):92–105. 18955434 10.1101/gr.082701.108 PMC2612969

[30] Quann K, Jing Y, Rigoutsos I. Post-transcriptional regulation of BRCA1 through its coding sequence by the miR-15/107 group of miRNAs. Front Genet. 2015;6:242. 26257769 10.3389/fgene.2015.00242 PMC4513244

[31] Wang C, Lin Y, Fu Y, et al. MiR-221-3p regulates the microvascular dysfunction in diabetic retinopathy by targeting TIMP3. Pflugers Arch. 2020 Nov;472(11):1607–1618. 32648125 10.1007/s00424-020-02432-y

[32] He S, Zhang W, Li X, et al. Oral squamous cell carcinoma (OSCC)-derived exosomal MiR-221 targets and regulates phosphoinositide-3-kinase regulatory subunit 1 (PIK3R1) to promote human umbilical vein endothelial cells migration and tube formation. Bioengineered. 2021 Dec;12(1):2164–2174. 34098850 10.1080/21655979.2021.1932222 PMC8806445

[33] Araya J, Tsubouchi K, Sato N, et al. PRKN-regulated mitophagy and cellular senescence during COPD pathogenesis. Autophagy. 2019 Mar;15(3):510–526. 30290714 10.1080/15548627.2018.1532259 PMC6351145

[34] Chen X, Li Y, Hua C, et al. Establishment of rapid risk assessment model for cigarette smoke extract exposure in chronic obstructive pulmonary disease. Toxicol Lett. 2019 Nov;316:10–19. 31476341 10.1016/j.toxlet.2019.08.020

[35] Vij N, Chandramani-Shivalingappa P, Van Westphal C, et al. Cigarette smoke-induced autophagy impairment accelerates lung aging, COPD-emphysema exacerbations and pathogenesis. Am J Physiol Cell Physiol. 2018 Jan 1;314(1):C73–c87. 27413169 10.1152/ajpcell.00110.2016 PMC5866380

[36] Livak KJ, Schmittgen TD. Analysis of relative gene expression data using real-time quantitative PCR and the 2(-Delta Delta C(T)) method. Methods. 2001 Dec;25(4):402–408. 11846609 10.1006/meth.2001.1262

[37] Cao Y, Liu Y, Ping F, et al. miR-200b/c attenuates lipopolysaccharide-induced early pulmonary fibrosis by targeting ZEB1/2 via p38 MAPK and TGF-β/smad3 signaling pathways. Lab Invest. 2018 Mar;98(3):339–359. 29200203 10.1038/labinvest.2017.123

[38] Shetty SK, Tiwari N, Marudamuthu AS, et al. p53 and miR-34a feedback promotes lung epithelial injury and pulmonary fibrosis. Am J Pathol. 2017 May;187(5):1016–1034. 28273432 10.1016/j.ajpath.2016.12.020 PMC5417006

[39] Zheng X, Zhao FC, Pang Y, et al. Downregulation of miR-221-3p contributes to IL-1β-induced cartilage degradation by directly targeting the SDF1/CXCR4 signaling pathway. J Mol Med (Berl). 2017 Jun;95(6):615–627. 28236026 10.1007/s00109-017-1516-6

[40] Yang Y, Li H, Ma Y, et al. MiR-221-3p is down-regulated in preeclampsia and affects trophoblast growth, invasion and migration partly via targeting thrombospondin 2. Biomed Pharmacother. 2019 Jan;109:127–134. 30396069 10.1016/j.biopha.2018.10.009

[41] Shen Y, Lu H, Song G. MiR-221-3p and miR-92a-3p enhances smoking-induced inflammation in COPD. J Clin Lab Anal. 2021 Jul;35(7):e23857. 34097306 10.1002/jcla.23857 PMC8274981

[42] Zhao J, Cui L, Sun J, et al. Notoginsenoside R1 alleviates oxidized low-density lipoprotein-induced apoptosis, inflammatory response, and oxidative stress in HUVECS through modulation of XIST/miR-221-3p/TRAF6 axis. Cell Signal. 2020 Sep 15;76:109781. 32947021 10.1016/j.cellsig.2020.109781

[43] Zhu L, Gong X, Gong J, et al. Notoginsenoside R1 upregulates miR-221-3p expression to alleviate ox-LDL-induced apoptosis, inflammation, and oxidative stress by inhibiting the TLR4/NF-κB pathway in HUVECs. Braz J Med Biol Res. 2020;53(6):e9346. 32401923 10.1590/1414-431X20209346 PMC7233198

[44] Rong J, Xu J, Liu Q, et al. Anti-inflammatory effect of up-regulated microRNA-221-3p on coronary heart disease via suppressing NLRP3/ASC/pro-caspase-1 inflammasome pathway activation. Cell Cycle. 2020 Jun;19(12):1478–1491. 32372677 10.1080/15384101.2020.1754562 PMC7469527

[45] Scully KM, Lahmy R, Signaevskaia L, et al. E47 governs the MYC-CDKN1B/p27(KIP1)-RB network to growth arrest PDA cells independent of CDKN2A/p16(INK4A) and wild-type p53. Cell Mol Gastroenterol Hepatol. 2018;6(2):181–198. 30003124 10.1016/j.jcmgh.2018.05.002 PMC6039985

[46] Whitcomb EA, Tsai YC, Basappa J, et al. Stabilization of p27(Kip1)/CDKN1B by UBCH7/UBE2L3 catalyzed ubiquitinylation: a new paradigm in cell-cycle control. Faseb J. 2019 Jan;33(1):1235–1247. 30113882 10.1096/fj.201800960R PMC6355086

[47] Wu DM, Wen X, Han XR, et al. MiR-142-3p enhances cell viability and inhibits apoptosis by targeting CDKN1B and TIMP3 following sciatic nerve injury. Cell Physiol Biochem. 2018;46(6):2347–2357. 29742504 10.1159/000489626

[48] Chen D, Wang X, Huang J, et al. CDKN1B mediates apoptosis of neuronal cells and inflammation induced by oxyhemoglobin via miR-502-5p after subarachnoid hemorrhage. J Mol Neurosci. 2020 Jul;70(7):1073–1080. 32152938 10.1007/s12031-020-01512-z

